# Understanding the multifaceted role of ABA signaling in orchestrating plant developmental transition

**DOI:** 10.1007/s44154-024-00203-8

**Published:** 2025-01-09

**Authors:** Yupeng Jiang, Shiyu Jiang, Lu Liu

**Affiliations:** Shanghai Collaborative Innovation Center of Agri-Seeds/Joint Center for Single Cell Biology, School of Agriculture and Biology, Shanghai Jiao Tong University, Shanghai, 200240 China

**Keywords:** ABA, Developmental transition, Germination, Seedling establishment, Flowering time, Dormancy

## Abstract

Abscisic acid (ABA), a pivotal plant hormone once primarily associated with stress response, is now increasingly acknowledged for its indispensable role in plant development. This comprehensive review delves into the multifaceted functions of ABA in regulating various aspects of plant growth and development. From inhibiting germination to orchestrating seedling establishment, flowering time, and dormancy induction, ABA emerges as a central player in shaping plant developmental transitions. Unraveling the intricate regulatory mechanisms governing the ABA signaling pathway provides valuable insights into how plants adapt to environmental challenges while effectively managing their growth and reproductive strategies. This expanding knowledge not only highlights the significance of ABA in plant biology but also has profound implications for enhancing agricultural practices.

## Introduction

Plants heavily rely on external environmental signals and internal developmental cues to orchestrate the timing of developmental transitions throughout their life cycle, governing key events such as germination, early seedling establishment, flowering time, and bud dormancy (Bäurle and Dean 2006; Chen et al. 2020; Kavi Kishor et al. 2022). Understanding the molecular mechanisms underlying these transitions is essential for agricultural productivity, ecological conservation, and adaptation to environmental changes.

Germination is the transition from the embryonic to the postembryonic mode of growth, setting the stage for subsequent developmental events (Ali et al. 2021; Bäurle and Dean 2006). Seedling establishment builds upon germination, as the seedling acquires resources and undergoes structural and physiological changes to establish itself as a mature plant. Seedling establishment is completed once the seedling attains photosynthetic proficiency, transitioning into an autotrophic state (Gommers and Monte 2018). After entering the seedling growing stage, plants receive signals from earlier developmental stages, environmental cues, and internal factors such as hormone balances, ensuring floral transition at the most optimized condition and maximizing reproductive success (Liu et al. 2021). Dormancy induction, on the other hand, represents a transition from active growth to a quiescent state, often triggered by environmental cues such as temperature changes or photoperiod shifts (Penfield 2017; Shu et al. 2016; Yang et al. 2021). All these developmental processes not only influence the subsequent stages but also integrate signals from proceeding and concurrent stages, orchestrating the growth and adaption of plants to the ever-changing environment in a coordinated manner.

Among the various signaling pathways involved, abscisic acid (ABA) signaling emerges as a central player, exerting multifaceted regulatory effects on plant development. ABA, known primarily for its role in stress responses, is increasingly recognized for its role in coordinating the timing of developmental transition (Ali et al. 2021; Chen et al. 2020; Kavi Kishor et al. 2022). ABA acts as a key regulator in modulating the activity and behavior of plant cells, often steering them toward a state of inactivity or dormancy. By modulating processes such as germination, seedling establishment, flowering time, and dormancy induction, ABA integrates internal developmental cues with external environmental signals, ensuring that plants respond appropriately to changing growth conditions. This review aims to delve into the intricate interplay between ABA signaling and the timing of developmental transitions in plants, shedding light on the underlying mechanisms and their implications for plant growth and adaptation.

## ABA signaling pathway

ABA is widely distributed throughout the plant kingdom, highlighting its fundamental importance in plant biology. The synthesis of ABA in plants using the carotenoid pathway initiated from a C_40_ precursor known as β-carotene. Key enzymes in ABA biosynthesis, including several 9-*cis*-epoxycarotenoid dioxygenase (*NCED*) genes, have been identified across various species (Chen et al. 2020). ABA is synthesized in response to environmental cues such as drought, salinity, and high temperatures, as well as during various developmental stages such as seed maturation and dormancy induction (Chen et al. 2020). Once synthesized, ABA acts systemically, orchestrating physiological responses across different tissues and organs. These coordinated actions of ABA ensure the adaption and survival of plants in dynamic environmental conditions (Kang et al. 2015; Qin et al. 2021; Waadt et al. 2014).

**Fig. 1:**
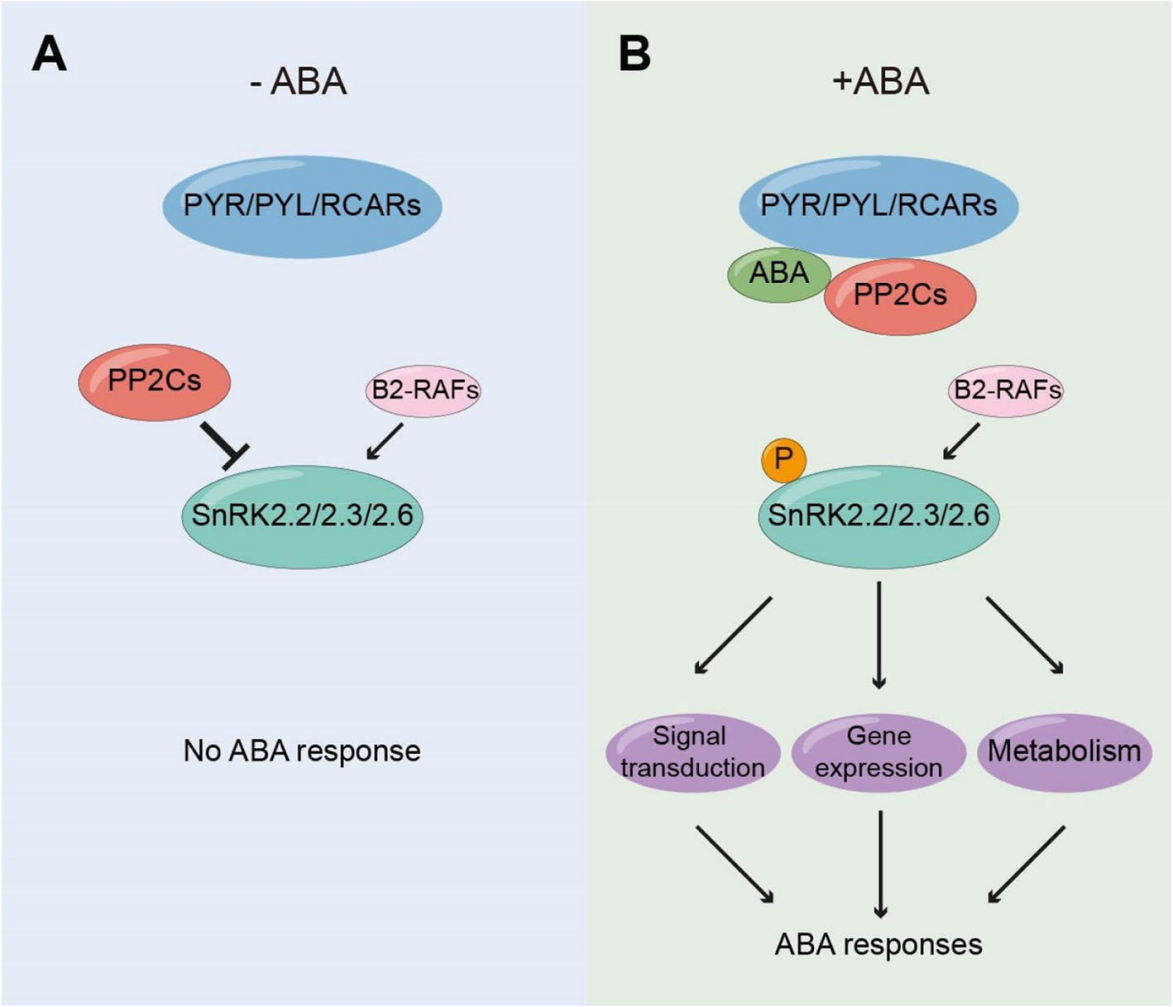
Main components in the core ABA signal transduction pathway. **A** In the absence of ABA, PP2Cs are active and suppress the activity of SnRK2.2/2.3/2.6 and downstream signaling pathways. **B** In the presence of ABA, PYR/PYL/RCARs bind to PP2Cs, releasing SnRK2.2/2.3/2.6 from suppression. The SnRK2.2/2.3/2.6 then become activated through autophosphorylation or phosphorylation by B2-RAFs and subsequently, activated SnRK2.2/2.3/2.6 trigger a cascade of downstream processes, including signaling transduction, gene expression, and metabolism, thereby initiating the ABA signaling pathway

Elevated levels of ABA trigger the activation of a central signaling pathway comprising three protein classes: the Pyrabactin Resistance (PYR)/PYR1-like (PYL)/Regulatory Component of ABA Receptor (RCAR) family, clade A Protein Phosphatase 2C (PP2C), and subclass III SNF1-related Protein Kinase 2s (SnRK2s) SnRK2.2, SnRK2.3, and SnRK2.6 (Cutler et al. 2010; Hubbard et al. 2010) (Fig. 1). In the absence of ABA, clade A PP2Cs are active and suppress the activity of subclass III SnRK2s and their downstream signaling pathways. However, upon exposure to ABA, the ABA-bound PYR/PYL/RCAR receptors undergo a structure alteration, engaging with PP2Cs and inhibiting their phosphatase activity. Consequently, subclass III SnRK2s are released from suppression and become activated through phosphorylation (Chen et al. 2020; Hubbard et al. 2010). The activation of subclass III SnRK2s has been traditionally thought to be attributed to self-activation through autophosphorylation following their release from the repression of PP2Cs. However, recent studies have identified two subfamilies of group B Raf-like kinases, B2-RAFs and B3-RAFs, that phosphorylate and activate these subclass III SnRK2s, facilitating their rapid phosphorylation and activation in response to ABA (Lin et al. 2021, 2020; Takahashi et al. 2020). High-ordered Arabidopsis mutants lacking multiple B2- and B3-RAFs exhibit ABA hyposensitivity. In these mutants, the activity of subclass III SnRK2s is significantly reduced under dehydration and ABA treatment conditions (Lin et al. 2021). B2-RAFs are constitutively active and further activate subclass III SnRK2s once PP2Cs are inhibited by ABA-bound receptors, thus playing a crucial role in ABA-dependent signaling. In contrast, B3-RAFs are activated under stress conditions in an ABA-independent manner (Soma et al. 2023). This dual mechanism ensures that SnRK2s can respond rapidly to both ABA signals and osmotic stress, highlighting a unique mechanism for the initiation and amplification of SnRK2s activation in ABA signaling and stress response in higher plants.

Serving as pivotal regulators within ABA signaling, activated subclass III SnRK2s phosphorylate downstream targets such as ABA Responsive Element Binding Proteins (AREBs)/ABRE Binding Factors (ABFs) gene subfamily members, thereby initiating cellular responses. This phosphorylation cascade subsequently triggers the transcriptional activation or repression of ABA-responsive genes involved in diverse developmental processes. By comparing the global changes in phosphopeptides between WT and *snrk2.2/2.3/2.6* triple mutant seedlings in response to ABA, unique peptides specifically phosphorylated by subclass III SnRK2s have been identified. Proteins containing these peptides are involved in various developmental processes, including flowering time regulation, RNA and DNA binding, signal transduction, metabolism, and epigenetic regulation, shedding light on the role of subclass III SnRK2s in transmitting ABA signaling to downstream targets (Kulik et al. 2011; Umezawa et al. 2013; Wang et al. 2013a). Moreover, the core components of the ABA signaling pathway, including PYL/PYR/RCAR receptors, PP2Cs, and SnRK2s, are tightly regulated, thus fine-tuning plant growth under suboptimal environmental conditions (Chen et al. 2020).

## ABA regulates key developmental transitions in plants

By inducing cellular quiescence, ABA helps plants cope with adverse conditions by slowing down growth and metabolic activity, thus preserving energy and mitigating damage. This induced cellular quiescence also coordinates plant developmental transition and morphogenesis, therefore modulating growth processes to adapt to changing environmental cues (Kavi Kishor et al. 2022).

### ABA and germination

The transition from embryo to seedling during germination represents a pivotal stage in the life cycle of plants, making the commencement of active growth and development. ABA is an essential repressor of seed germination (Sajeev et al. 2024). The endosperm, a single cell layer surrounding the embryo, synthesizes and continuously releases ABA toward the embryo, which process represents a crucial aspect of seed development and germination regulation (Kang et al. 2015; Lee et al. 2010b). During seed germination, several components of the ABA signaling cascade are involved, working together to mediate the effects of ABA during seed germination. ABA receptors PYLs lead to the inhibition of PP2Cs, allowing the activation of SnRK2.2/2.3. These kinases then phosphorylate and activate the basic leucine zipper (bZIP) transcription factor ABA INSENSITIVE 5 (ABI5), along with a B3-domain transcription factor ABSCISIC ACID INSENSITIVE 3 (ABI3), to regulate the expression of genes involved in maintaining seed dormancy and inhibiting germination (Fujii et al. 2007; Lopez-Molina et al. 2002; Zhao et al. 2020). Among these components, ABI5 plays a key role in the regulation of this ABA-mediated developmental checkpoint (Finkelstein 1994; Finkelstein and Lynch 2000; Lopez-Molina et al. 2002).

**Fig. 2:**
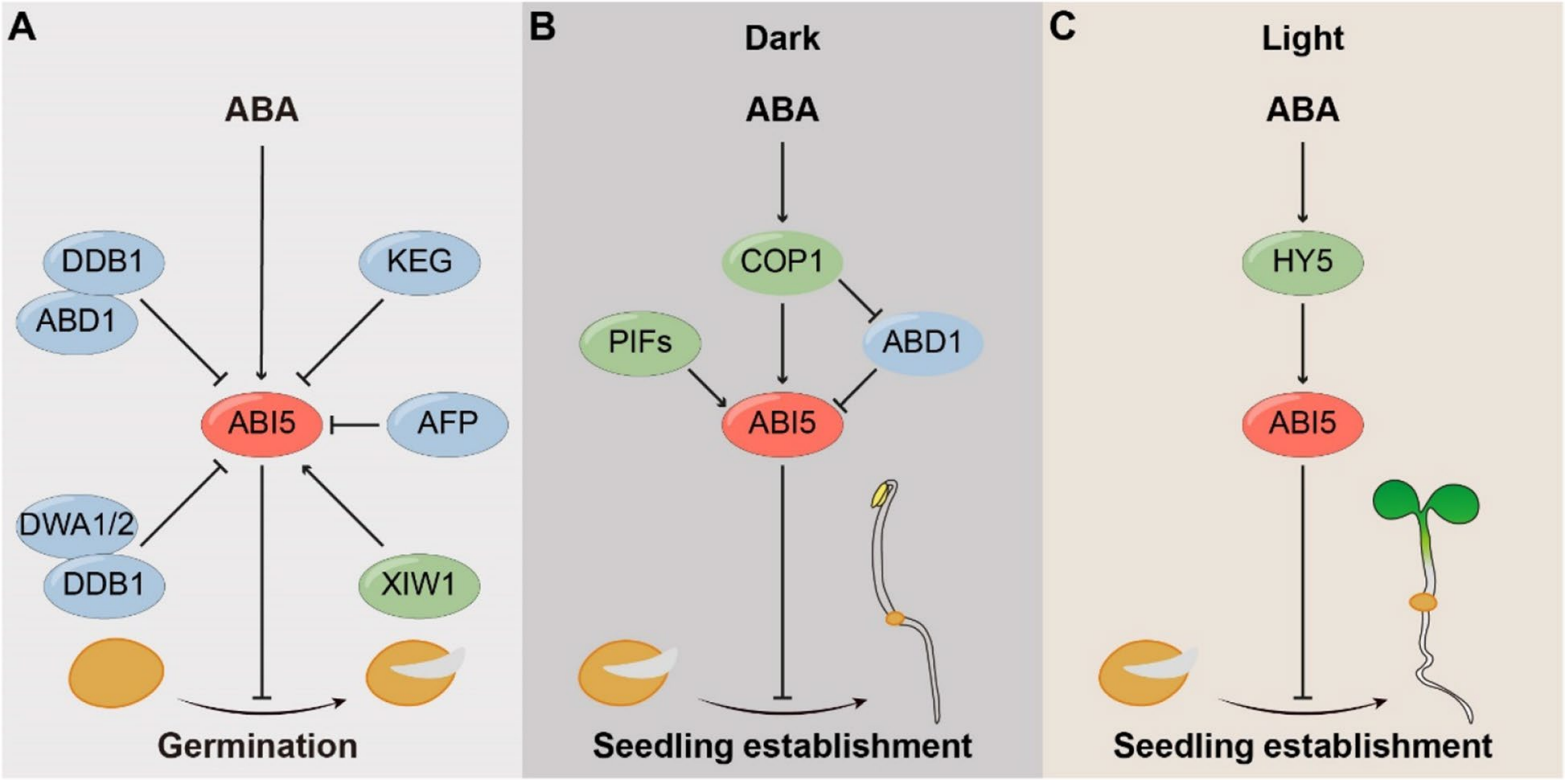
ABI5 functions as a central regulator of ABA-mediated inhibition of germination and seedling establishment. **A** During seed germination, KEG and AFP negatively regulate the ABI5 protein level. ABD1 interacts with DDB1 to promote ABI5 degradation. Additionally, DWA1 and DWA2 interact with DDB1 to facilitate ABI5 degradation. **B** During seedling establishment in darkness, COP1 positively regulates ABA signaling by facilitating the binding of ABI5 to its target promoters and mediates the turnover of ABD1, a substrate receptor of E3 ligase, which targets ABI5 for degradation. PIFs activate *ABI5* transcription by directly binding to the *ABI5* promoter. **C** During seedling establishment in light, HY5 directly activates the expression of *ABI5* in the presence of ABA. Green indicates positive regulators of ABI5, while blue indicates negative regulators of ABI5

ABI5 acts as a central regulator of ABA-mediated inhibition of germination. The accumulation and activity of ABI5 are induced within a short interval following stratification by ABA at both transcriptional and translational levels (Lopez-Molina et al. 2001; Yu et al. 2015) (Fig. 2A). By acting as a transcriptional repressor, ABI5 helps to maintain seed dormancy and prevent germination in unfavorable environmental conditions. Once the condition becomes conducive for germination, ABA levels decrease, leading to the degradation of ABI5 by the ubiquitin 26S proteasome proteolytic pathway and subsequently activation of germination-promoting genes (Brocard et al. 2002; Lopez-Molina et al. 2003; Yu et al. 2015).

ABI5 is a transcription factor that is predominantly localized in the nucleus. Several nuclear-localized E3 ubiquitin ligases to mediate ABI5 degradation are also identified. Two DWD genes, DWD hypersensitive to ABA1 (DWA1) and DWA2, have been implicated in ABA hypersensitivity. These genes interact with ABI5 and DDB1, an adaptor for the CULLIN4-based E3 ligase complex, thus facilitating ABI5 degradation in the nucleus (Lee et al. 2010a). ABA-hypersensitive DCAF1 (ABD1) is another substrate receptor in CULLIN4-based E3 ubiquitin ligases. Similarly, ABD1 interacts with ABI5 and DDB1 in the nucleus, negatively regulating ABA signaling by promoting ABI5 degradation (Seo et al. 2014). ABI five binding protein (AFP) targets ABI5 for ubiquitin-based degradation in nuclear bodies, further regulating ABI5 protein stability (Lopez-Molina et al. 2003). Conversely, XPO1-interacting WD40 protein 1 (XIW1) positively regulates the seedling ABA response by interacting with ABI5 in the nucleus and maintaining its stability (Xu et al. 2019).

KEEP ON GOING (KEG), a RING E3 ligase, has been identified as a negative regulator of ABA signaling and is required to maintain low levels of ABI5. ABI5 shuttles between the cytoplasm and nucleus, as it contains both a potential nuclear export signal (NES) and three potential nuclear localization signals (NLSs) (Liu and Stone 2013). KEG localizes to the trans-Golgi network and early endosomes (TGN/EE), where it interacts with ABI5, and promotes the turnover of ABI5 in the cytoplasm. Consequently, *keg* mutant plants exhibit elevated ABI5 levels, rendering them hypersensitive to ABA treatment (Gu and Innes 2012; Liu and Stone 2013; Stone et al. 2006). Collectively, the precise degradation of ABI5 in both the cytoplasm and nucleus fine-tunes ABA signaling, influencing seed germination. In addition to protein ubiquitination, ABI5 activity is precisely regulated by phosphorylation, dephosphorylation, and sumoylation (Fujii et al. 2007; Hu and Yu 2014; Nakashima et al. 2009; Yu et al. 2015). Furthermore, multiple transcription factors regulate *ABI5* expression at the transcriptional level (Skubacz et al. 2016). Together, these mechanisms ensure the fine-tuning of the ABI5 signaling, highlighting its critical role in ABA-mediated seed germination.

### ABA and seedling establishment

Seedling establishment is a critical phase in the life cycle of the plant, encompassing the period from seed germination to the point where the young plants become self-sustaining and capable of continued growth. One of the dramatic changes for developing seedlings is exposure to light, initiating a process known as photomorphogenesis. In the presence of light, seedlings undergo de-etiolation, characterized by the expansion of cotyledons, the formation of chloroplasts, and the cessation of hypocotyl elongation (Liu et al. 2020). ABA plays a crucial role in regulating post-germination growth arrest, particularly in response to adverse environmental conditions. Once seeds have germinated and seedlings have emerged, they are susceptible to various stresses such as drought, salinity, and high temperatures. ABA acts as a signaling molecule that helps plants cope with these stress signals and reversibly arrest growth during this narrow developmental window after germination and before the onset of vegetative growth, thus often having antagonistic effects with light on seedling development (Lopez-Molina et al. 2001). This interplay between ABA and light helps balance growth and growth arrest in seedlings (Yadukrishnan and Datta 2021). Under optimal light conditions, ABA levels may decrease, promoting growth and development. Conversely, under stress conditions such as drought, ABA levels increase to enhance stress tolerance. Notably, in dark conditions, ABA-mediated seedling growth inhibition is enhanced, highlighting the dynamic interplay between light and ABA during early seedling establishment (Yadukrishnan and Datta 2021; Yadukrishnan et al. 2020).

ABI5 also serves as a central hub for these interactions between ABA and light, transducing ABA signals during post-germination seedling establishment (Fig. 2B and 2C). The protein level and activity of ABI5 are quickly induced during a limited developmental time window poststratification, helping plants sustainably resistant to extended stress conditions (Lopez-Molina et al. 2001). Once activated, ABI5 directly regulates several growth-related genes, helping the plant respond to environmental cues and physiological needs. By influencing phosphorus uptake, chlorophyll production, photosynthetic efficiency, and cell wall integrity, ABI5 fine-tunes the seedlings transition from dependent seeds to independent photosynthetic organisms (Carles et al. 2002; Cheng et al. 2014; Huang et al. 2017; Yang et al. 2023).

Light modulates the ABA signaling pathway by regulating ABI5 at both transcriptional and posttranslational levels (Fig. 2B). CONSTITUTIVELY PHOTOMORPHOGENIC 1 (COP1) is a highly conserved protein and functions as a RING E3 ubiquitin ligase to mediate the degradation of multiple proteins involved in light perception (Han et al. 2020). COP1 positively regulates ABA signaling during seedling growth in the dark and promotes seedling development arrest by facilitating the binding of ABI5 on its target promoters (Yadukrishnan et al. 2020). Furthermore, COP1 promotes ABA-induced ABI5 protein stability post-translationally in the dark by mediating the turnover of ABD1, a substrate receptor of E3 ligase, in response to ABA via the 26S proteasome pathway (Peng et al. 2022). Consistently, the *cop1* mutant shows an ABA hyposensitive phenotype during postgermination seedling development (Peng et al. 2022; Yadukrishnan et al. 2020). Moreover, the expression of *ABI5* is directly regulated by Phytochrome-Interacting Factors (PIFs), a group of basic helix-loop-helix transcription factors that are accumulated in the dark and repress seedling photomorphogenesis (Qi et al. 2020). Thus, PIFs represent a molecular hub that integrates light signals to regulate ABA signaling and post-germination seedling growth. Furthermore, ELONGATED HYPOCOTYL 5 (HY5), another key regulator of light signaling, is also shown to promote ABA signaling by directly activating *ABI5* (Chen et al. 2008) (Fig. 2C). Overall, the interplay between light and ABA signaling ultimately shapes the plant postgermination growth.

### ABA and flowering time

**Fig. 3:**
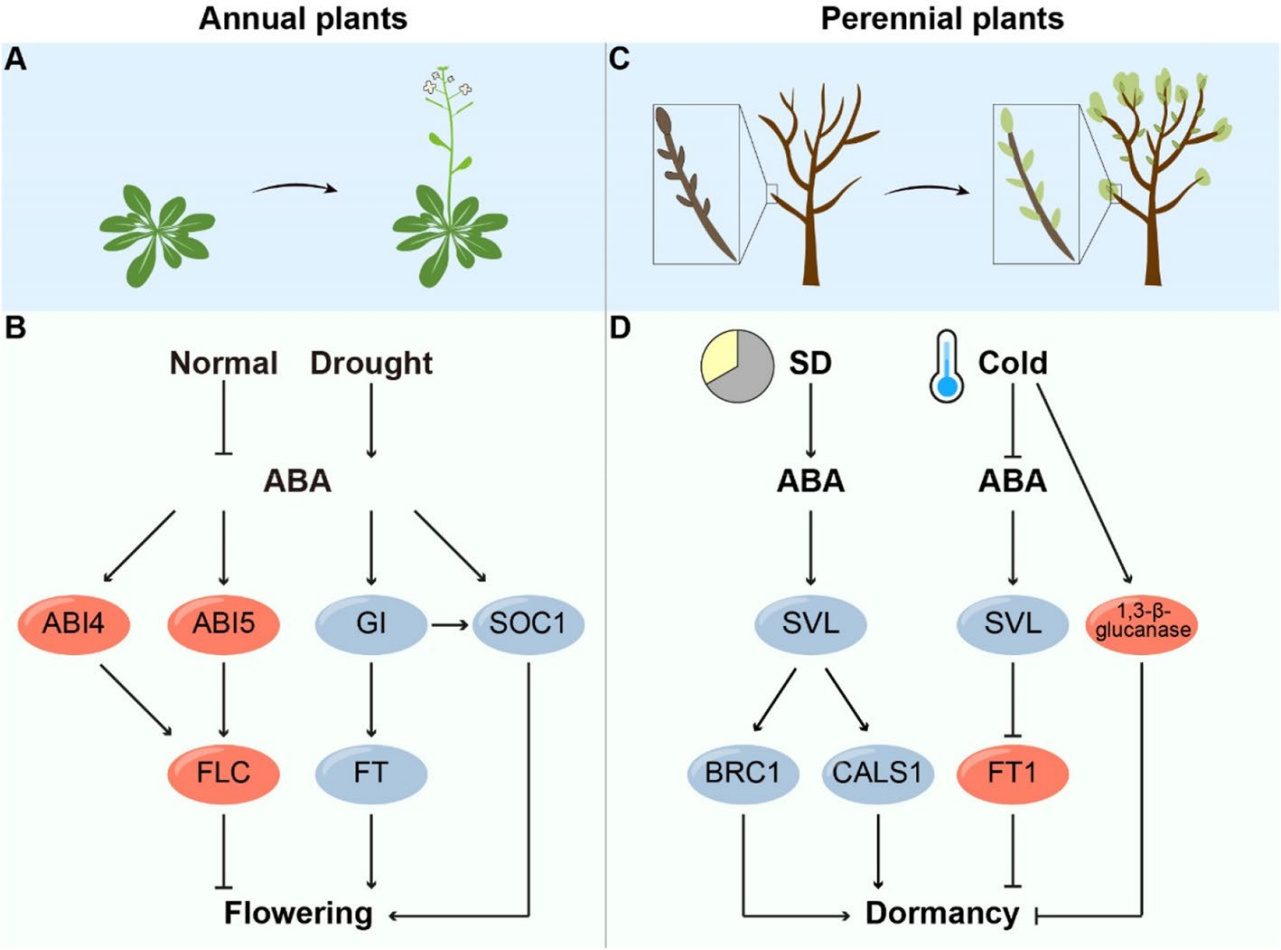
ABA plays different roles in annual and perennial plants. **A** Annual plants complete their life cycles within a single grown season. This process is characterized by a transition from vegetative growth to the reproductive phase. **B** In annual plants, ABA signaling activates the transcription factors *ABI4* and *ABI5*. ABI4 and ABI5 promote the expression of *FLC*, thereby delaying the flowering transition. ABA also stimulates the expression of *GI*, which subsequently upregulates the expression of *FT* and *SOC1*, promoting flowering. ABA also activates *SOC1* to promote flowering. **C** Perennial plants maintain a longer lifespan and ensure successful reproduction through the precise regulation of bud dormancy and its release in response to changing external environments, such as varying temperatures and photoperiods. **D** In perennial plants, short photoperiods elevate ABA levels, which in turn stimulate the expression of *SVL*. SVL induces *CALS1* and *BRC1* to promote dormancy. Chilling temperatures trigger a decline in ABA levels within the buds and suppress the expression of *SVL*, leading to the activation of *FT1* expression. FT1 then promotes dormancy release. Additionally, chilling temperatures stimulate the upregulation of 1,3-β-glucanase genes to break dormancy. Red indicates positive regulators, while blue indicates negative regulators

Plants intricately coordinate the transition to flowering by integrating a multitude of internal cues and external environmental signals, all aimed at maximizing plant reproductive success. Among the key players in this complex process is gibberellin (GA), which significantly influences flowering time by integrating environmental cues and flowering genetic pathways (Bao et al. 2020). ABA functions antagonistically with GA, regulating various biological processes such as seed dormancy, germination, seedling growth, and responses to environmental cues (Liu and Hou 2018). Notably, ABA signaling has garnered increasing attention for its role in modulating floral transition (Shu et al. 2018) (Fig. 3A).

ABA is considered a floral suppressor. The inhibitory role of ABA in floral transition has been demonstrated through studies on ABA-deficient mutants. These mutants impaired in ABA production display altered flowering time (Domagalska et al. 2010). Furthermore, plants with application of exogenous ABA significantly delay floral transition in Arabidopsis, suggesting that ABA might be an endogenous hormone regulating flowering time (Zhang 2014; Wang et al. 2013b).

The transcription factor ABI5 plays a critical role in negatively regulating flowering time by directly activating the expression of *FLOWERING LOCUS C* (*FLC*), a key floral integrator that delays flowering transition (Wang et al. 2013b) (Fig. 3B). Phosphorylation of ABI5 by SnRK2s is essential for its function in governing flowering time. Interestingly, in mutants lacking *SnRK2.2/2.3/2.6*, despite the presence of ABA, the inhibitory effect of ABI5 on floral transition is abolished (Wang et al. 2013b). Additionally, *SnRK2.2/2.3/2.6* triple mutant flowers earlier than WT, particularly under SD growth conditions. The expression of several flowering-related genes is altered, suggesting that SnRK2 might mediate the phosphorylation status of other regulators involved in flowering time, thereby influencing plant reproductive growth (Wang et al. 2013a). ABA INSENSITIVE 2 (ABI2), a phosphatase, dephosphorylates ABI5, thereby reducing its ability to induce *FLC* expression and promote flowering (Ali et al. 2024). Furthermore, FCS-like Zinc Finger Protein 13 (FLZ13) interacts with ABI5 and collaborates with it to enhance the expression of *FLC*, emphasizing the crucial roles of ABI5 in modulating flowering time (Li et al. 2024; Yang et al. 2023). Another player in this regulatory network is ABA INSENSITIVE 4 (ABI4), an AP2/ERF domain-containing transcription factor involved in the ABA signaling pathway. The *abi4* mutant shows an early flowering phenotype, whereas the overexpression of *ABI4* delays floral transition by directly promoting *FLC* expression (Shu et al. 2015). These findings underscore the intricate role of ABI4 and ABI5 in modulating flowering time, highlighting the complex relationship between ABA signaling and flowering time regulation.

Although several pieces of evidence demonstrate that ABA delays flowering, it also has been reported that ABA promotes floral transition, especially under environmental stress conditions, such as drought (Shu et al. 2018). Drought Escape (DE) represents a traditional adaptive mechanism wherein plants undergo accelerated development to complete their life cycle before an impending drought event. The significance of early flowering time and a condensed vegetative phase plays a crucial role in reducing vulnerability to dehydration during sensitive stages of plant growth like flowering and post-anthesis grain filling. This enables plants to set seeds before severe stress conditions threaten their survival (Shavrukov et al. 2017). ABA is indispensable for the DE response and exerts a positive influence on flowering time, particularly under long-day conditions (LDs) (Fig. 3B). In LDs, the DE-triggered ABA signaling cascade stimulates the expression of *GIGANTEA* (*GI*), which subsequently upregulates the expression of *FLOWERING LOCUS T* (*FT*) and *SUPPRESSOR OF OVEREXPRESSION OF CONSTANS1* (*SOC1*) (Riboni et al. 2013, 2016). Moreover, under drought conditions, GI interacts with ENHANCED EM LEVEL (EEL), a bZIP transcription factor, to activate the rate-limiting ABA biosynthesis enzyme *NCED3*, thereby enhancing plant drought tolerance (Baek et al. 2020). The ABA signaling pathway additionally plays a pivotal role in enabling plants to evade drought stress, facilitated by ABF transcription factors. Notably, ABF3 and ABF4 govern flowering time in response to drought by directly orchestrating the transcriptional activity of the floral integrator SOC1 (Hwang et al. 2019). Taken together, all these regulatory mechanisms aid in the overall completion of plant life cycles under drought conditions, ensuring adaption and survival in challenging environments.

The roles of ABA in regulating flowering time are multifaceted, involving complex interactions with molecular components and environmental cues. Elevated ABA levels, often associated with high levels of *ABI4* and *ABI5*, can act as a suppressor of flowering by prolonging the vegetative phase or impeding the transition to reproductive growth. Conversely, the positive impact of ABA on flowering time is typically observed in response to stress signals like drought. It has been suggested that plants possess distinct sensors capable of discerning the source of ABA accumulation, whether from internal developmental cues or external environmental factors, therefore subsequently activating different responsiveness (Shu et al. 2018). Overall, the dual nature of ABA in flowering time regulation underscores its diverse roles in plant physiology and adaptation to environmental stresses.

### ABA and bud dormancy

Unlike annual plants that complete their life cycle within a single growing season, perennial plants undergo seasonal growth patterns, that show repeated cycles of growth, reproduction, and dormancy over multiple years. Bud dormancy serves as a critical survival strategy employed by perennial plants to endure adverse environmental conditions, particularly during harsh winter seasons. During dormancy, plants enter a state of suspended growth and metabolic activity in their buds (Ruttink et al. 2007; Yang et al. 2021). This period of dormancy allows plants to survive unfavorable conditions and thrive in challenging environments, ensuring their long-term persistence and reproductive success (Fig. 3C).

Plants rely on light and temperature as crucial environmental signals to discern seasonal variations and transitions. As day length decreases during autumn, plants interpret these changes in photoperiod as a signal to initiate dormancy. Interestingly, the regulatory mechanism known as CONSTANS (CO)-FT regulatory cascade, which governs flowering time in the annual plant Arabidopsis, also plays a role in controlling the growth cessation and bud dormancy in woody perennials like aspen trees (Böhlenius et al. 2006). Upon sensing short-day (SD) signals, the expression of *FT2*, a pivotal gene in the CO-FT pathway, is promptly suppressed (Hsu et al. 2011). This suppression of *FT2* subsequently triggers the downregulation of *Like-APERALA1* (*LAP1*), a MADS-box transcription factor that is highly similar to the Arabidopsis floral meristem identity gene *AP1*. *LAP1*, in response, activates the expression of *AINTEGUMENTA-like 1* (*AIL1*), which, in turn, regulates the cell cycle by activating important cell cycle genes like *CYCD3.2* (Azeez et al. 2014; Karlberg et al. 2011). Moreover, the SD-induced downregulation of *LAP1* alleviates its repression effect on *BRANCHED1* (*BRC1*), a known negative regulator of lateral bud outgrowth and bud break inhibition (Aguilar-Martínez et al. 2007; Maurya et al. 2020; Singh et al. 2018). Notably, BRC1 interacts with FT2, reinforcing the repression of SD on growth cessation (Maurya et al. 2020). This intricate regulatory network highlights how plants fine-tune their responses to environmental signals to modulate growth and development.

Short photoperiods also elevate ABA levels within hybrid aspen buds, triggering the transition to dormancy in the shoot apex (Fig. 3D). In hybrid aspen plants expressing the dominant-negative *abi1-1* allele, which reduces ABA responses, dormancy establishment is impaired. However, wild-type and *abi1-1* plants exhibit growth cessation and apical bud formation under SD conditions, suggesting that attenuated ABA responses compromise dormancy establishment rather than growth cessation (Tylewicz et al. 2018). The increased ABA level in SDs stimulates the expression of *SVP-like* (*SVL*), an ortholog of *SHORT VEGETATIVE PHASE* (*SVP*) in Arabidopsis, which suppresses flowering. SVL further induces the callose synthase enzyme *CALLOSE SYNTHASE 1* (*CALS1*), involved in callose deposition and closure of plasmodesmata (PDs), while repressing GA2 oxidase genes in GA biosynthesis, thereby inhibiting cell-to-cell communication and reducing the potential for growth reinitiation (Singh et al. 2019). Additionally, SVL modulates ABA levels and signaling by activating the key ABA biosynthesis enzyme *NCED3* and the ABA receptor *PYL1/2*, forming a feedback loop to enhance plant responses to ABA (Singh et al. 2018). SVL also directly activates the expression of *BRC1*, inhibiting bud activity and increasing ABA levels in the apices, thereby promoting dormancy establishment (González-Grandío et al. 2017; Singh et al. 2018). Interestingly, in the annual plant Arabidopsis, despite not following a yearly growth cycle, SVP has also been demonstrated to regulate the ABA pathway (Wang et al. 2018). In Arabidopsis, SVP is induced by drought stress and subsequently modulates the expression of genes involved in ABA catabolism, such as *CYP707A1*, *CYP707A3*, and *AtBG1*. This regulatory activity leads to elevated cellular ABA levels and makes plants more resistant to water deficit (Wang et al. 2018). These discoveries suggest that modulation of cellular ABA levels by *SVP* and *SVP*-like genes is a significant mechanism for terrestrial plants to manage adverse environmental conditions.

Once dormancy is established, buds become unresponsive to growth signals, ensuring their protection during winter. However, breaking dormancy requires prolonged exposure to cold temperatures, a process known as the chilling requirement, which is crucial for plants to resume growth in the spring. This requirement maintains bud dormancy throughout winter, safeguarding them from potential frost damage and other environmental stress. As temperatures gradually decrease, factors that sustain bud dormancy are repressed. This reduction in temperature also triggers a decline in ABA levels within the buds and suppresses the expression of *SVL*, leading to the activation of *FT1* expression and promoting GA biosynthesis (Singh et al. 2018). Moreover, chilling temperature stimulates the upregulation of cell wall 1,3-β-glucanase genes, facilitating the turnover of callose at PDs (Fig. 3D). This action promotes the opening of cell-to-cell communication, enabling the movement of FT signals into the buds, thus initiating shoot elongation and morphogenesis (Rinne et al. 2011; Tylewicz et al. 2018). The cyclic pattern of dormancy and growth is indispensable for the successful development of perennial plants, ensuring their resilience and reproductive success in fluctuating seasonal environments.

### ABA and seed dormancy

Seed dormancy refers to a state in which seeds are unable to germinate even under favorable environmental conditions. This dormancy leads to the interruption of the continuous growth of the plant embryo and results in a dormant seed (Raz et al. 2001). Seed dormancy is a vital adaptive mechanism that allows seed plants to delay germination until environmental conditions are favorable for successful establishment and growth. This strategy increases the likelihood of survival and successful establishment.

ABA is a key hormone involved in maintaining seed dormancy. By modulating the expression of various genes involved in seed dormancy, ABA promotes the entry of seeds into a dormant state and inhibits germination, helping the seed prepare for potential stress and environmental changes (Bentsink and Koornneef 2008). ABI3 is a key transcription factor that plays a pivotal role in regulating seed dormancy and inhibiting seed germination mediated by ABA (Bentsink and Koornneef 2008; Liu et al. 2013; Rohde et al. 2000). When the embryo matures, ABI3 is required to lower water potential in seeds enough to inhibit germination and activate other developmental progress involved in late seed development. Early in the seed maturation phase, ABI3 promotes embryo dormancy in Arabidopsis. Mutations in this gene can result in premature germination of the seeds (Raz et al. 2001). Furthermore, severe mutants in *abi3* significantly change cell behavior during embryogenesis and display an array of seed-specific developmental defects, suggesting that ABI3 may have a broader role beyond just ABA signaling (Rohde et al. 2000). In maize, the *Viviparous 1* (*Vp1*) gene, an ortholog of ABI3 in Arabidopsis, plays a crucial role as a significant component of ABA signaling that influences seed dormancy (McCarty et al. 1991). Ectopic expression of maize *Vp1* in wheat has been shown to enhance both seed dormancy and tolerance to preharvest sprouting (Huang et al. 2012). *FUSCA3* (*FUS3*), *LEAFY COTYLEDON 1* (*LEC1*), and *LEC2* are the other three key regulators that are critical for mid- and late-seed development. *FUS3* and *LEC1* encode transcription factors containing the conserved B3 DNA binding domain, whereas *LEC1* encodes a CCAAT binding transcription factor containing a HAP3 subunit. The *abi3*, *lec1*, *lec2*, and *fus3* are all critical for proper seed maturation and share several common phenotypes such as their reduced dormancy and lower expression of seed storage proteins (Bentsink and Koornneef 2008). The shared phenotypes among these mutants underscore the importance of *ABI3*, *LEC1*, *LEC2*, and *FUS3* in ensuring proper seed development and viability.

In addition to general mutants that affect seed dormancy, alterations in ABA biosynthesis also play a significant role in regulating this process. Mutations in genes involved in ABA biosynthesis can lead to changes in ABA concentration within the seed, directly influencing its ability to enter and maintain a dormant state. Key genes such as *ABA DEFICIENT 1 (ABA1)* and *ABA2* are critical for ABA production. Mutations in these genes can disrupt normal ABA synthesis, resulting in phenotypes characterized by reduced dormancy and altered seed development (Léon-Kloosterziel et al. 1996). Additionally, *NCED* genes are essential for a key regulatory step in ABA biosynthesis. The triple mutant *nced5 nced6 nced9* exhibits significant seed dormancy defects, further demonstrating that endogenous ABA is crucial for maintaining seed dormancy (Frey et al. 2012; Lefebvre et al. 2006). Taken together, these findings indicate that changes in ABA levels and signaling pathways can determine whether seeds remain dormant or germinate, significantly impacting their survival and establishment in diverse environmental conditions.

## Conclusions and perspective

Plants are continuously exposed to a wide range of environmental conditions, requiring them to adapt to changing environments. ABA, traditionally acknowledged for its involvement in stress responses, is gaining prominence for its pivotal role in orchestrating the timing of developmental transitions in plants, including the regulation of seed germination, seedling establishment, flowering time, and dormancy induction, thereby facilitating adaptation to changing surroundings. In response to environmental cues or endogenous developmental signals, ABA level increases, leading to the inhibition of cell growth and the induction of dormancy in various plant tissues, therefore coordinating the growth and development of plants.

In addition to its critical role in regulating developmental transition, ABA also serves as a key regulator of plant organ development. Effective soil exploration by roots relies on lateral root formation, which is essential for accessing water and nutrients and is also critical for crop improvement and food security. The adaptive mechanism of exrobranching regulates LR formation based on soil moisture, promoting root branching when root tips encounter moist soil (Orman-Ligeza et al. 2018). This response is ABA-dependent and is conserved across both eudicot and monocot species. In response to transient water deficits, the ABA signaling pathway is activated, requiring PYR/PYL/RCAR-dependent signaling in Arabidopsis (Orman-Ligeza et al. 2018). Furthermore, PYL8 is necessary for initiating genes that restore LR growth under rehydration conditions in Arabidopsis (Zhao et al. 2014). Under optimal moisture, roots transport auxin and water symplastically to initiate LR formation. However, under limited moisture, roots depend on internal water reserves, and ABA reduces plasmodesmata aperture, restricting auxin transport and halting LR formation. Upon rehydration, ABA levels drop, auxin movement resumes, and LR formation continues. These mechanisms help roots adapt to fluctuating soil moisture levels (Mehra et al. 2022). In water-deficient conditions, several regulatory modules coordinate ABA and auxin signaling to modulate LR development, allowing plants to adapt their root branching patterns to heterogeneous soil moisture (Bao et al. 2014; Mehra et al. 2022; Zhang et al. 2023).

ABA also promotes xylem differentiation, facilitating the specialization of cells to form the water-conducting tissues of vascular plants. ABA enhances xylem differentiation by activating VASCULAR-RELATED NAC DOMAIN (VND) transcription factor. Specifically, VND2 and VND3 accelerate the differentiation of metaxylem cells, while VND7 promotes the transformation of metaxylem into protoxylem-like structures (Ramachandran et al. 2021). This direct activation by ABA highlights a connection between ABA signaling and xylem development. Under osmotic stress, ABA also induces the expression of microRNAs miR165a/166b, which in turn repress Class III HD-ZIP transcription factors such as PHABULOSA (PHB) (Bloch et al. 2019; Miyashima et al. 2011; Ramachandran et al. 2018). This repression is essential for proper xylem patterning and for coordinating the differentiation of various root tissues. Additionally, ABA suppresses stem cell differentiation in the primary root and promotes the quiescence of main root growth (Luo et al. 2014; Zhang et al. 2010). Furthermore, ABA can transmit dehydration signals from roots long distances to the shoot to modulate stomatal closure (Takahashi et al. 2018). Overall, ABA emerges as a central regulator of plant organ development, optimizing water use and enhancing stress tolerance to enable plants to adapt and survive in unfavorable growth conditions.

The significant involvement of ABA in regulating various aspects of plant growth and development underscores its importance as a key player in adaptation processes. By modulating responses to environmental stimuli and fine-tuning developmental pathways, ABA ensures that plants thrive in diverse habitats and successfully navigate fluctuating environmental conditions. Thus, the multifaceted functions of ABA highlight its indispensable role in plant adaptation and survival in ever-changing environments.

## Data Availability

Not applicable.

## Abbreviations

ABA: Abscisic acid
NCED: 9-*cis*-epoxycarotenoid dioxygenase
PYR: Pyrabactin Resistance
PYL: PYR1-like
RCAR: Regulatory Component of ABA Receptor
PP2C: Protein Phosphatase 2C
SnRK2: SNF1-related Protein Kinase 2
AREB: ABA Responsive Element Binding Protein
ABF: ABRE Binding Factor
ABI3: ABA INSENSITIVE 3
ABI5: ABA INSENSITIVE 5
bZIP: Basic leucine zipper
KEG: KEEP ON GOING
NES: Nuclear export signal
NLS: Nuclear localization signal
TGN: Trans-Golgi network
EE: Early endosomes
DWA1: DWD hypersensitive to ABA1
ABD1: ABA-hypersensitive DCAF1
AFP: ABI five binding protein
XIW1: XPO1-interacting WD40 protein 1
COP1: CONSTITUTIVELY PHOTOMORPHOGENIC 1
HY5: ELONGATED HYPOCOTYL 5
GA: Gibberellin
FLC: FLOWERING LOCUS C
FLZ13: FCS-like Zinc Finger Protein 13
DE: Drought Escape
LD: Long-day condition
GI: GIGANTEA
FT: FLOWERING LOCUS T
SOC1: SUPPRESSOR OF OVEREXPRESSION OF CONSTANS1
EEL: ENHANCED EM LEVEL
CO: CONSTANS
LAP1: Like-APERALA1
AIL1: AINTEGUMENTA-like 1
BRC1: BRANCHED1
SVP: SHORT VEGETATIVE PHASE
SVL: SVP-like
CALS1: CALLOSE SYNTHASE 1
PDs: Plasmodesmata
Vp1: Viviparous 1
FUS3: FUSCA3
LEC1: LEAFY COTYLEDON 1
ABA1: ABA DEFICIENT 1
VND: VASCULAR-RELATED NAC DOMAIN
PHB: PHABULOSA
